# Vancomycin-resistant enterococci in Spanish hospitals: the experience of official network for the surveillance of resistant microorganisms RedLabRA, 2023–2024

**DOI:** 10.3389/fpubh.2026.1966902

**Published:** 2026-10-29

**Authors:** Javier E. Cañada-García, Sara Pérez de Madrid, Estela Giménez Quiles, María Pilar Ortega-Lafont, María Dolores Tirado-Balaguer, Luis López-Urrutia, Esther Viedma-Moreno, Marta Hernández-García, Nieves Larrosa-Escartín, Julia Serrano-Lobo, Ignasi Roca, Jordi Càmara, Diego García Martínez-de-Artola, Ana Isabel López-Calleja, Paloma Merino Amador, Cristina Seral, Lorena López-Cerero, Fernando Lázaro-Perona, Ferran Navarro, Fernando González-Candelas, María Pérez-Vázquez, Jesús Oteo-Iglesias, the Spanish RedLabRA Network Study Group

**Affiliations:** 1 Laboratorio de Referencia e Investigación en Resistencia a Antibióticos e Infecciones relacionadas con la Asistencia Sanitaria, Centro Nacional de Microbiología, Instituto de Salud Carlos III, Madrid, Spain; 2 Laboratory Network for the Surveillance of Resistant Microorganisms (RedLabRA), Madrid, Spain; 3 CIBER de Enfermedades Infecciosas (CIBERINFEC), Instituto de Salud Carlos III, Madrid, Spain; 4 Servicio de Microbiología, Hospital Universitario de Guadalajara, Guadalajara, Spain; 5 Servicio de Microbiología, Hospital Clínico Universitario de Valencia, Valencia, Spain; 6 Complejo Asistencial Universitario de Burgos, Burgos, Spain; 7 Servicio de Microbiología, Hospital General Universitario de Castellón, Castellón de La Plana, Spain; 8 Servicio de Microbiología, Hospital Universitario Río Hortega, Valladolid, Spain; 9 Servicio de Microbiología, Hospital Universitario 12 de Octubre, Madrid, Spain; 10 Servicio de Microbiología, Hospital Universitario Ramón y Cajal, Madrid, Spain; 11 Servicio de Microbiología, Hospital Universitario Vall d’Hebron, Barcelona, Spain; 12 Servicio de Microbiología, Hospital Universitario Gregorio Marañón, Madrid, Spain; 13 Servicio de Microbiología, Hospital Clinic, Barcelona, Spain; 14 Servicio de Microbiología Clínica, Hospital Universitari de Bellvitge-IDIBELL, L'Hospitalet de Llobregat, Barcelona, Spain; 15 CIBER de Enfermedades Respiratorias (CIBERES), Instituto de Salud Carlos III, Madrid, Spain; 16 Servicio de Microbiología, Hospital Universitario Nuestra Señora de Candelaria, Santa Cruz de Tenerife, Spain; 17 Servicio de Microbiología, Hospital Universitario Miguel Servet, IIS Aragón, Zaragoza, Spain; 18 Servicio de Microbiología, Hospital Universitario Clínico San Carlos, Madrid, Spain; 19 Instituto de Investigación Sanitaria Hospital Clínico San Carlos (IdISSC), Madrid, Spain; 20 Servicio de Microbiología, Hospital Universitario Lozano Blesa, Zaragoza, Spain; 21 Servicio de Microbiología, Hospital Universitario Virgen Macarena, Sevilla, Spain; 22 Servicio de Microbiología, Hospital Universitario La Paz, Madrid, Spain; 23 Servicio de Microbiología, Hospital Universitario Santa Creu i Sant Pau, Barcelona, Spain; 24 Unidad Mixta de Investigación en Infección y Salud Pública, FISABIO, Universidad de Valencia - I2SysBio, Valencia, Spain; 25 CIBER de Epidemiología y Salud Pública (CIBERESP), Instituto de Salud Carlos III, Madrid, Spain

**Keywords:** enterococci, *Enterococcus faecalis*, *Enterococcus faecium*, linezolid, RedLabRA, surveillance, vancomycin resistant, whole genome sequencing

## Abstract

**Objectives:**

RedLabRA is a network of microbiology laboratories for the genomic surveillance of antibiotic resistance. The aim of this study was to describe the actions undertaken by RedLabRA in response to an increase of vancomycin-resistant enterococci (VRE) in some Spanish regions.

**Methods:**

RedLabRA designed a VRE surveillance organized in two phases: (1) a questionnaire to assess risk perception of VRE among the network’s Level 2 laboratories (LN2), and (2) collection of cases detected between April 2023 and March 2024, followed by WGS of a sample of vancomycin-resistant *Enterococcus faecium* (VREfm) isolates to investigate interregional dissemination. WGS was performed using the Illumina platform; MLST (PubMLST.org scheme), cgMLST and phylogenetic relationships (Ridom SeqSphere+ v3.5.0), and resistome (Resfinder 4.7.2) were analysed.

**Results:**

Nine of the 17 autonomous communities defined a priority scenario of VRE. 31 LN2 reported 1,958 VRE cases from 86 hospitals across 12 autonomous communities and the autonomous city Ceuta. The predominant species was *E. faecium* (97.1%). 317 VREfm isolates were included for WGS: 211 (66.6%) carried the *van*A gene, 90 (28.4%) *van*B, and 16 (5%) both genes. ST117 (34.7%), ST80 (27.1%), or ST1421 (25.6%) predominated. Using cgMLST (1,423 genes, cutoff of 5 alleles), 45 VREfm clusters were identified, of which eight involved at least three hospitals and were inter-regional. 51 (16.1%) isolates carried linezolid resistance mechanisms, mainly *optr*A+*cfr*D.

**Conclusion:**

VREfm had a highly uneven impact across Spanish regions. The inter-regional presence of VREfm isolates with high genetic similarity belonging to ST1421, ST117, and ST80 clones was detected. The emergence of *E. faecium* isolates carrying genes conferring resistance to vancomycin and linezolid requires ongoing surveillance.

## Introduction

Antimicrobial resistance (AMR) is a dynamic and rapidly evolving public health problem that significantly limits the therapeutic options for infectious diseases. It is difficult to determine the current burden of antimicrobial resistance (AMR) due to the predominance of heterogeneous, fragmented, and retrospective data sources. These limitations raise concerns regarding the implementation of an agile and effective surveillance. An active genomic surveillance allows a better understanding of bacterial dynamics and to establish strain/mechanism associations (1), facts that are crucial to carry out fast interventions in public health (2).

The Spanish National Plan against Antibiotic Resistance (PRAN) is a strategic and action plan aimed to reduce the spread of AMR and its clinical and public health impact (3). The PRAN is coordinated by the Agencia Española del Medicamento y Productos Sanitarios (AEMPS) and has the participation of all the autonomous communities, nine Spanish Ministries, and more than 70 scientific societies, collegiate organizations, professional associations and universities.

Promoted by the PRAN, the Spanish Network of Laboratories for the Surveillance of Resistant Microorganisms (RedLabRA) was established as a nationally coordinated and interconnected network of microbiology laboratories. Its purpose is to support the diagnosis and molecular characterization of bacterial lineages and antimicrobial resistance mechanisms, as well as to monitor their evolutionary trends and patterns of interregional dissemination. RedLabRA is an official surveillance system with the participation of all Spanish autonomous communities through regional sub-networks coordinated by laboratories specifically designated by the regional health authorities (4, 5).

Life-threatening nosocomial infections caused by multidrug-resistant *Enterococcus faecalis* and *Enterococcus faecium* have emerged over recent decades, posing a major challenge to both patients and healthcare professionals (6). Both are responsible for a wide range of infections, including bloodstream infections, urinary tract infections (UTIs), and infective endocarditis, particularly among immunocompromised patients (7).

The intrinsic and acquired resistance mechanisms of these pathogens frequently limit available therapeutic options. Specifically, vancomycin-resistant *E. faecium* (VREfm) emerged in the 1980’s, most likely through the horizontal acquisition of the *van*A and *van*B gene clusters and subsequent spread across multiple continents with increasing prevalence (8–11). In particular, the hospital-adapted lineage belonging to clonal complex 17 (CC17) has been identified as a globally disseminated high-risk lineage associated with severe nosocomial outbreaks (12). This lineage has demonstrated a remarkable capacity to acquire and maintain antimicrobial resistance genes.

Despite vancomycin and linezolid being regarded as last-resort therapeutic options for the treatment of multidrug-resistant enterococcal infections, resistance to these antibiotics has increased in recent years. Linezolid resistance is primarily mediated by mutations in the 23S rRNA gene or by the acquisition of transferable resistance genes, including *cfrD*, *optrA*, and *poxtA* (13, 14).

The coordinating laboratories of RedLabRA in the autonomous communities of Catalonia, Aragon, and the Valencian Community alerted the network coordination team to a significant increase in cases of vancomycin-resistant enterococci (VRE) in their regions during the last months of 2023 and the first months of 2024. This situation prompted a proactive initiative by the network coordination team to assess the current national status of this issue.

The aim of this study was to describe the actions undertaken by RedLabRA in response to the observed increase in VRE. These actions included: (i) the distribution of a questionnaire (22 March 2024) to participating laboratories to obtain their subjective assessment of the VRE situation in their autonomous communies; and (ii) the molecular characterization, by whole-genome sequencing (WGS), of a sample of isolates collected between April 2023 and March 2024, with the ultimate aim of assessing potential interhospital and interregional dissemination.

## Materials and methods

### RedlabRA structure

The RedLabRA framework is based on a three-tier organization of all clinical microbiology laboratories in Spain. Level 1 laboratories (LN1) comprise all public and private clinical microbiology laboratories within the National Health System and constitute the cornerstone of the Network. At a minimum, these laboratories must be able to phenotypically detect or suspect the resistant pathogens and resistance mechanisms under surveillance. LN1 laboratories refer suspected isolates to Level 2 laboratories (LN2), which perform rapid molecular characterization of resistance mechanisms and circulating lineages and are also able to investigate outbreaks. LN2 laboratories are designated by each autonomous community and have the capacity to perform whole-genome sequencing (WGS). The Level 3 laboratory (LN3) corresponds to the National Centre for Microbiology of the Instituto de Salud Carlos III (CNM-ISCIII), although additional laboratories may be designated by the Network for specific diagnostic purposes. In addition to the functions performed by LN2 laboratories, the LN3 is responsible for coordinating the Network, developing protocols, and conducting nationwide comparative and evolutionary studies based on WGS of a sample of isolates from clones circulating in the autonomous communities. Furthermore, the CNM-ISCIII may provide support for Level 2 activities whenever deemed necessary (4, 5).

All 17 Spanish autonomous communities participate in RedLabRA and have designated LN2 for the coordination of the surveillance subnetworks in their respective geographical areas. In total, there are 40 LN2 throughout Spain, ranging from 1 to 8 per autonomous community.

### Study design and participating hospitals

A questionnaire regarding cases of VRE (March 2024) was distributed to the LN2 laboratories to obtain their self-reported perception of the priority of this public health issue in their autonomous communities: critical, high, medium-high, medium, or low priority (Supplementary material-Appendix 2).

As a result of the information provided by the questionnaire, basic information was collected for all VRE cases detected by the network between April 2023 and March 2024. Finally, from the total number of cases recorded, a sample of VREfm was selected for WGS to investigate potential interhospital and interregional dissemination. The selection criteria were as follows: (i) when the sequence type (ST) and the resistance mechanism was available one isolate per vancomycin-resistance mechanism (*van*A/*van*B), ST, and hospital was selected; and (ii) for hospitals where the ST was unknown, isolates were selected based on epidemiological and microbiological criteria, prioritizing the selection of isolates from different departments or with different microbiological profiles. The selection criteria were established to ensure the greatest possible diversity in terms of geographical areas, clones and resistance mechanisms, while simultaneously avoiding overrepresentation caused by in-hospitals outbreaks or endemics. Due to this approach, the obtained results does not allow drawing general conclusions about the total national prevalence of clones and mechanisms.

For this study, a case of VRE was a patient infected or colonized by an *E. faecium* (*Efm*) or *E. faecalis* strain with a vancomycin MIC >4 mg/L, according to EUCAST guidelines (15). Only the first isolate per patient was considered, regardless of the isolation sample. Vancomycin susceptibility was determined by microdilution by participating LN2 using their routine methodology.

Some of the terms used in this study were defined as:

- Colonization: the presence and multiplication of microorganisms in a host without causing tissue damage or symptoms.
- Infection: the presence and multiplication of microorganisms in a host that cause invasion of tissues and generate symptoms, and inflammatory or immune reaction.
- Invasive infection: infection in which bacteria invade sterile body site or specimen, such as blood, cerebrospinal fluid, synovial fluid, peritoneal fluid or a normally sterile tissue, potentially causing systemic disease.
- Bacteremia: detection of viable bacteria in the bloodstream, usually demonstrated by a positive blood culture.
- Outbreak-associated cases: infections or colonizations that are epidemiologically linked to a recognized outbreak, typically involving the same or closely related bacterial strain.

### Whole-genome sequencing of bacterial isolates

WGS was performed by the CNM-ISCIII using the Illumina platform. Raw sequence data were submitted to the European Nucleotide Archive (PRJEB123727). A joint bioinformatics analysis was carried out on both the sequences generated by the LN2 laboratories and submitted to the network, as well as those obtained by the CNM in its role as the LN3 reference laboratory.

Raw reads quality was assessed using FASTQC, and reads were trimmed to remove adapters and low-quality bases using fastp (16). Genome assemblies were generated with Unicycler v0.4.8 (17), and assembly quality was evaluated using QUAST^1^. Bacterial species identification was confirmed by similarity analysis against reference genomes using KmerFinder (18).

Sequence types (STs) were determined using the MLST schemes and databases available at PubMLST.org (19) through ARIBA v2.6.2 (20). cgMLST complex types (CTs) were assigned using the cgMLST.org nomenclature server according to the species-specific allelic distance threshold (<=20 allelic differences for *Efm*). CTs function as additional unique identifiers to facilitate human communication.

Minimum spanning trees based on core-genome MLST (cgMLST) profiles were generated using Ridom SeqSphere+ v3.5.0, incorporating pairwise genetic distances calculated with MEGA X v10.0.5 (21). For *Efm*, a cgMLST scheme comprising 1,423 loci available in Ridom SeqSphere^2^ and developed by de Been et al. (22) was applied. Interhospital clusters were investigated using cgMLST and a hierarchical clustering approach, defining clusters as groups in which all pairwise comparisons differed by no more than five alleles, thereby ensuring the identification of compact clusters (pairwise missing values have been ignored). Although the original validation study of this scheme proposed a threshold of ≤20 allelic differences to define closely related *Efm* isolates, this threshold was intended primarily for broad cluster delineation rather than as definitive evidence of recent transmission (22). Therefore, to increase discriminatory power and minimise the inclusion of genetically similar but epidemiologically unrelated isolates, a more stringent threshold of ≤5 allelic differences was applied in the present study to define isolates as highly genetically related. This conservative threshold is supported by previous genomic investigations of VREfm. In a Swedish VREfm outbreak investigation, isolates belonging to a documented transmission cluster differed by 0–5 cgMLST alleles (23). More recently, a nationwide Swiss genomic survey using the same 1,423-locus *Efm* cgMLST scheme defined cgMLST clusters as isolates differing by ≤5 loci, explicitly based on experience from VREfm investigations (24).

Allelic distances were calculated from the allele-difference matrix, and agglomerative hierarchical clustering was performed using the distance, complete linkage, and fcluster functions from the scipy.cluster.hierarchy module^3^.

Resistome analysis was performed using ARIBA v2.6.2 (20) against the ResFinder database (version 22-03-2024) (25) and LRE-Finder for the identification of mutations associated with linezolid resistance with a cut-off of 10% mutations in position 2,576 and /or 2,505 (26).

### Statistical analysis

The difference between the frequency of STs, antibiotic resistance genes, and sample types detected in this study were evaluated using the Fisher’s exact test using GraphPad Prism (version 11.0.0; GraphPad Software, Inc., San Diego, CA, United States). *p*-values of less than 0.05 were considered statistically significant.

## Results

### Responses of the LN2 laboratories to the questionnaire

Twelve of the 17 Spanish autonomous communities, together with the autonomous city of Ceuta, reported VRE cases between January 2023 and March 2024. Nine of them detected an increase in the number of cases, with two, three, two and two autonomous communities considering critical, high, medium-high and medium priorities, respectively. Conversely, eight autonomous communities and the two Spanish autonomous cities classified this health problem as low priority (Figure 1).

**Figure 1:**
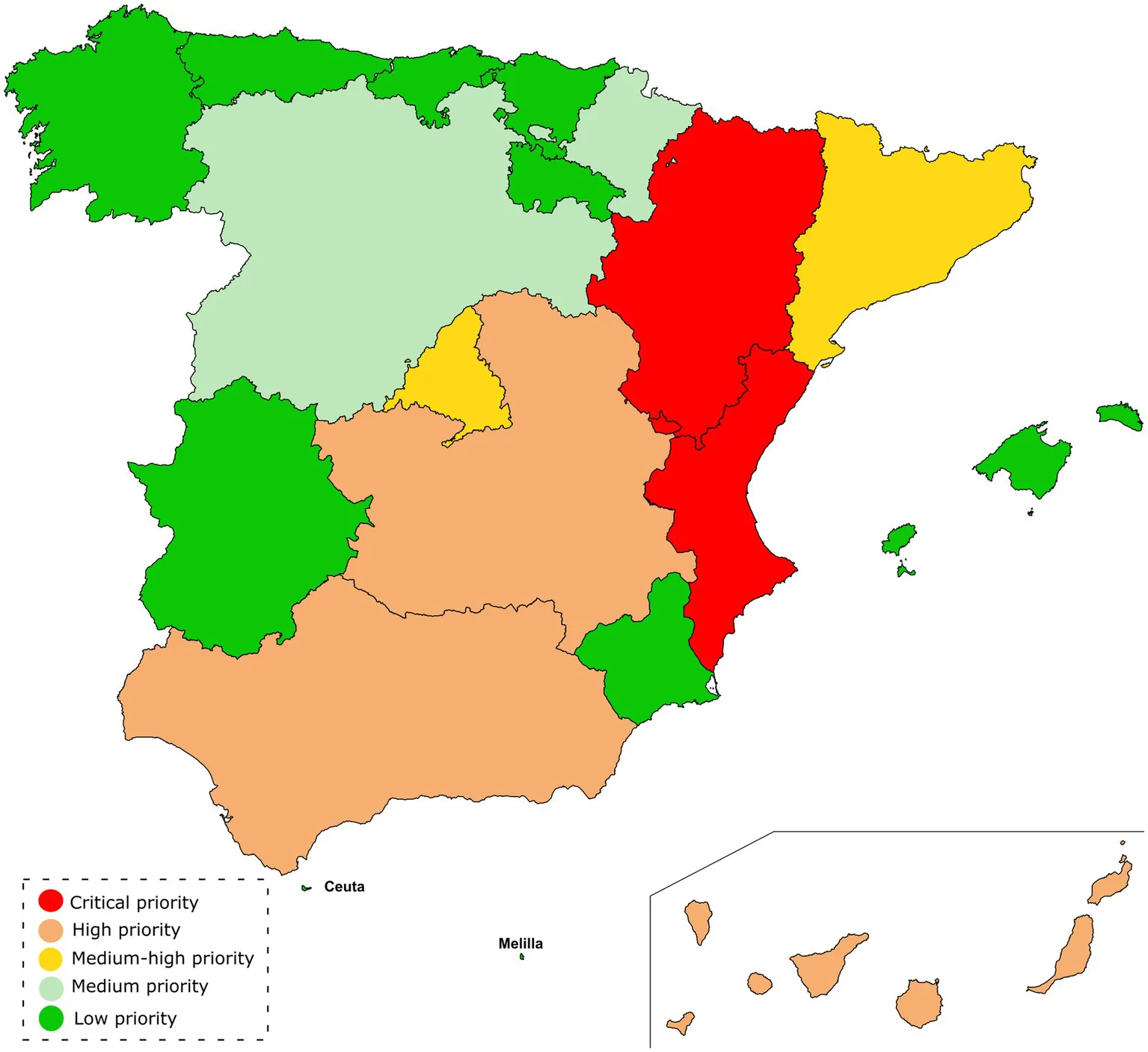
Self-reported perception of the priority of vancomycin resistance in enterococci among Level 2 RedLabRA laboratories, by Spanish autonomous community (January 2023–March 2024).

This questionnaire was conducted to understand the perceptions of LN2 in different autonomous communities regarding this resistance problem. Since it was found that more than half considered it a priority problem of medium to critical priority, a subsequent field study was designed for case collection.

### Participating hospitals and total cases reported

A total of 31 of the 40 Spanish LN2 communicated VRE cases in the regional subnetworks that coordinate. Globally the VRE cases were 1,958 diagnosed in 86 hospitals across 12 autonomous communities and the autonomous city of Ceuta (range per hospital: 1–249 cases). Despite the broad population coverage provided by RedLabRA, the absence of detection in a particular region does not necessarily imply the absence of cases. The autonomous communities reporting the highest numbers of cases were Madrid (446), Comunidad Valenciana (427), Castilla-León (323), Cataluña (310), and Castilla-La Mancha (295) (Table 1).

**Table 1:** Number of vancomycin-resistant *Enterococcus faecium* isolates detected between April 2023 and March 2024, by Autonomous Community.

Autonomous Community	N° of Hospitals	VRE	VREfm				
			Total	Sequenced isolates	*van*A (among sequenced isolates)	*van*B (among sequenced isolates)	*van*A+*van*B (among sequenced isolates)
Comunidad de Madrid	14	446	442	93	79	14	0
Comunidad Valenciana	8	427	423	47	17	20	10
Castilla y León	11	323	290	30	1	29	0
Cataluña	13	310	307	59	49	10	0
Castilla-La Mancha	7	295	291	41	32	3	6
Aragón	10	59	56	14	8	6	0
Islas Canarias	7	49	47	15	10	5	0
Andalucía	4	29	29	9	8	1	0
País Vasco	7	15	13	6	5	1	0
Murcia	2	2	2	2	2	0	0
Principado de Asturias	1	1	1	1	0	1	0
Ceuta	1	1	0	0	0	0	0
Galicia	1	1	0	0	0	0	0
Cantabria	0	0	0	0	0	0	0
Comunidad Foral de Navarra	0	0	0	0	0	0	0
Extremadura	0	0	0	0	0	0	0
Islas Baleares	0	0	0	0	0	0	0
La Rioja	0	0	0	0	0	0	0
Melilla	0	0	0	0	0	0	0
Total	86	1,958	1,901	317	211	90	16

VRE, vancomycin-resistant enterococci, including *E. faecium* plus *E. faecalis*; VREfm, vancomycin-resistant *E. faecium*.

Among the 1,812 cases for which specimen source information was available (missing data: 146), 127 (7%) were causing invasive infections, including 97 blood cultures. Most reported isolates were obtained from rectal swabs/stool samples (1,012, 55.8%), urine (316; 17.4%), and abscesses/wounds (316, 17.4%).

Of the 1,824 cases with available demographic information (missing data: 134), 57% (1,039) occurred in males and 43% (785) in females. Most cases affected patients were older than 65 years (66.2%, 1,196/1,808). The predominant species was *Efm*, accounting for 1,901 cases (97.1%).

### Efm isolates selected for WGS analysis

According to defined selection criteria, a sample of 317 Efm isolates [178, (56.2%) infections, 71 of them invasive infections, and 139 (43.8%) colonizations] was included for WGS to investigate potential interhospital and interregional transmission events (Table 1).

Among the sequenced Efm isolates, 211 (66.6%) carried the *van*A gene, 90 (28.4%) carried *van*B, and 16 (5%) harbored both resistance genes.

### Sequence types and genotypes

A total of 19 different sequence types (STs) were detected (mean: 16.7 isolates per ST; range: 1–110 isolates per ST). However, 277 (87.4%) of the isolates belonged to three STs: ST117 (110 isolates; 34.7%), ST80 (86 isolates; 27.1%), or ST1421 (81 isolates, 25.6%) (Figures 2A–C). ST1421 was significantly more frequent in infection (33.7%) than in colonization isolates (15.1%) (OR = 2.86, IC (95%) = 1.63–5.0, *p* = 0.0002), while ST80 was more frequent in colonization (33.1%) than in infection isolates (22.5%) (OR = 0.59, IC (95%) = 0.36–0.97, *p* = 0.04).

**Figure 2:**
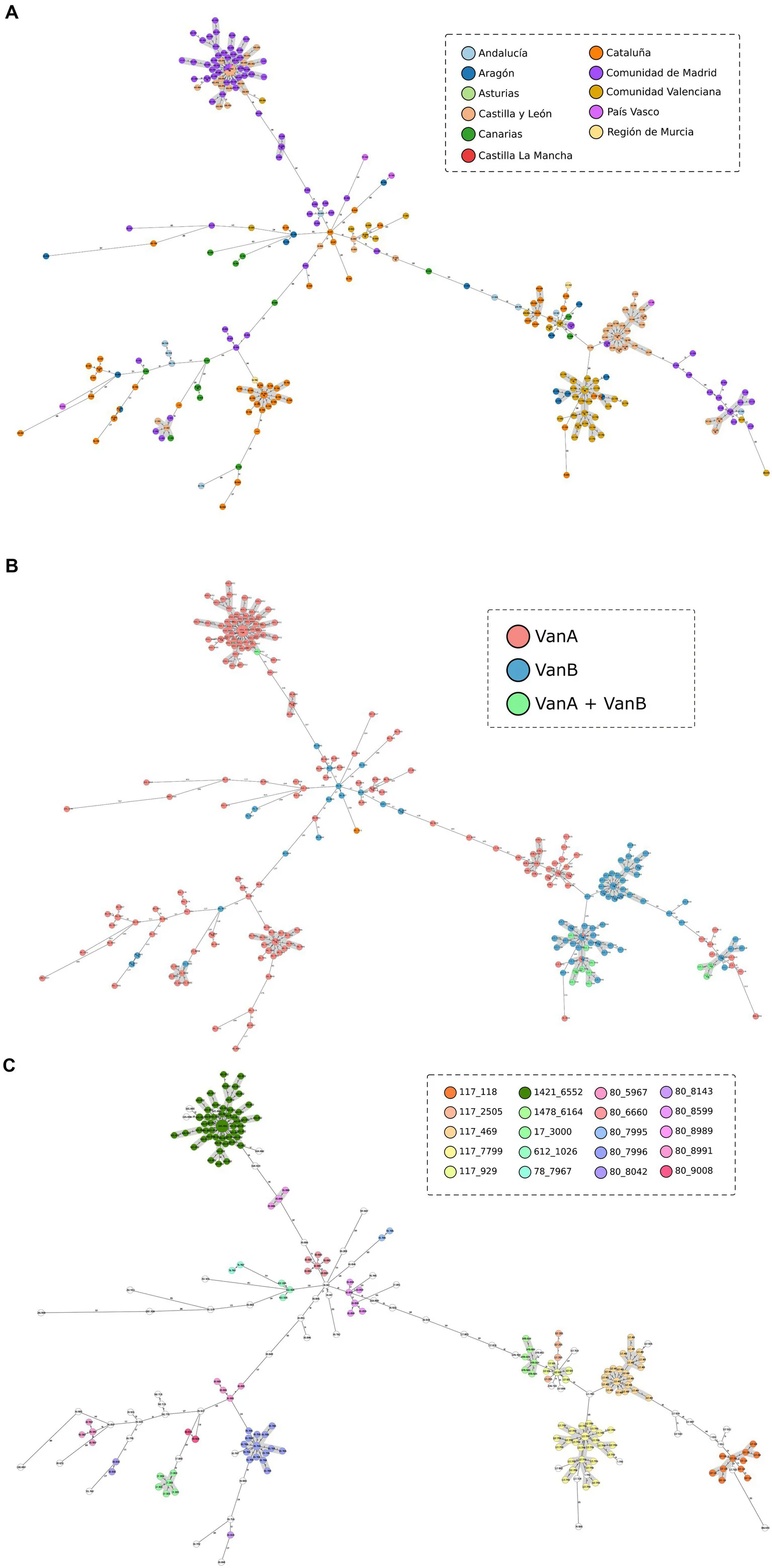
**(A–C)** Population structure of vancomycin-resistant *Enterococcus faecium*: minimum spanning tree. Genetic distances based on cgMLST for 1,423 genes are shown. The shading colour of the circles indicates the Autonomous Communities of origin of the isolate in image **A**, the gene responsible for vancomycin resistance in image **B**, and the sequence type and complex type (ST_CT) assigned by Ridom Seqsphere software in image **C**. The number inside the circle indicates the ST, CT and the number following the asterisk indicates the number of isolates. Numbers on the branches indicate allelic distances; branch lengths are not to scale.

ST1421 showed a strong association with the *van*A genotype (98.8%), statistically significant compared to the other non-ST1421 isolates (OR = 100.7, IC (95%) = 6.2–1643.8, *p* < 0.001) and was detected in hospitals in Castilla-La Mancha, Madrid, and País Vasco. ST80 was associated with *van*A (80.2%) and *van*B (19.8%) genotypes, whereas ST117 harbored *van*B (62.7%), *van*A (23.6%), and, interestingly, *van*A+*van*B (13.6%) genotypes. ST117 and ST80 exhibited a wide geographical distribution, being identified in 10 and 11 autonomous communities, respectively, while ST1421 was in central Spain, mainly affecting the autonomous communities of Madrid and Castilla-La Mancha.

The *van*A+*van*B genotype (*n* = 16) was statistically predominant in ST117 (16.7%) versus the other non-ST117 isolates (0.48%) (OR = 386.2, IC (95%) = 45.3–3295.9, *p* < 0.0001), with an equitable distribution between isolates from infections (8/178; 4.5%) and colonizations (8/139; 5.8%). This genotype was mainly detected in the Comunidad Valenciana (62.5%) and Castilla-La Mancha (37.5%).

### Clusters of isolates identified by cgMLST analysis (≤5 allele differences)

Using cgMLST with a cutoff of 5 alleles, 45 VREfm clusters were identified, of which 14 involved at least three hospitals. These clusters were predominantly associated with ST1421/*van*A/*van*A+*van*B, ST117/*van*A+*van*B, ST80/*van*A, ST17/*van*A, and ST1478/*van*A; eight clusters encompassed isolates originating from different regions. (Table 2).

**Table 2:** Interhospital clusters of vancomycin-resistant *Enterococcus faecium* isolates affecting three or more hospitals detected by cgMLST (≤5 alleles difference).

Clusters	ST_CT	N° isolates	Vancomycin resistance genes	N° Hospitals	Autonomous communities
GEnt0001	ST1421_6552	53	*van*A/*van*A+*van*B	14	Castilla-La Mancha, Comunidad de Madrid, País Vasco
GEnt0002	ST117_7799	15	vanA/*van*A+*van*B	7	Comunidad Valenciana, Aragón, Cataluña, Asturias
GEnt0003	ST80_7996	14	*van*A	5	Cataluña
GEnt0004	ST117_469	14	*van*B	6	Castilla y León, Comunidad de Madrid
GEnt0005	ST117_469	7	*van*B	4	Castilla y León, Castilla-La Mancha
GEnt0014	ST117_2505	4	*van*A	4	Cataluña, Región de Murcia
GEnt0006	ST117_118	7	*van*B/*van*A+*van*B	3	Castilla-La Mancha, Comunidad de Madrid
GEnt0012	ST80_5967	3	*van*A	3	Cataluña
GEnt0017	ST17_3000	3	*van*A	3	Castilla-La Mancha, Comunidad de Madrid
GEnt0021	ST1478_6164	3	*van*A	3	Cataluña
GEnt0022	ST117_929	3	*van*A/*van*A+*van*B	3	Comunidad Valenciana
GEnt0023	ST117_929	3	*van*A/*van*A+*van*B	3	Comunidad Valenciana, Cataluña
GEnt0024	ST117_118	3	*van*B	3	Comunidad de Madrid
GEnt0025	ST117_779	3	*van*B	3	Aragón

ST_CT, sequence type_complex_type.

The clusters affecting the greatest number of autonomous communities/hospitals were GEnt0001, comprising 53 isolates of ST1421 and involving 14 hospitals across 3 autonomous communities; all isolates except one had the *van*A genotype; and GEnt0002, comprising 15 ST117 isolates from seven hospitals across four autonomous communities (Table 2; Figure 3). The autonomous communities with the highest number of detected clusters were Cataluña, Madrid, Castilla-La Mancha and Comunidad Valenciana.

**Figure 3:**
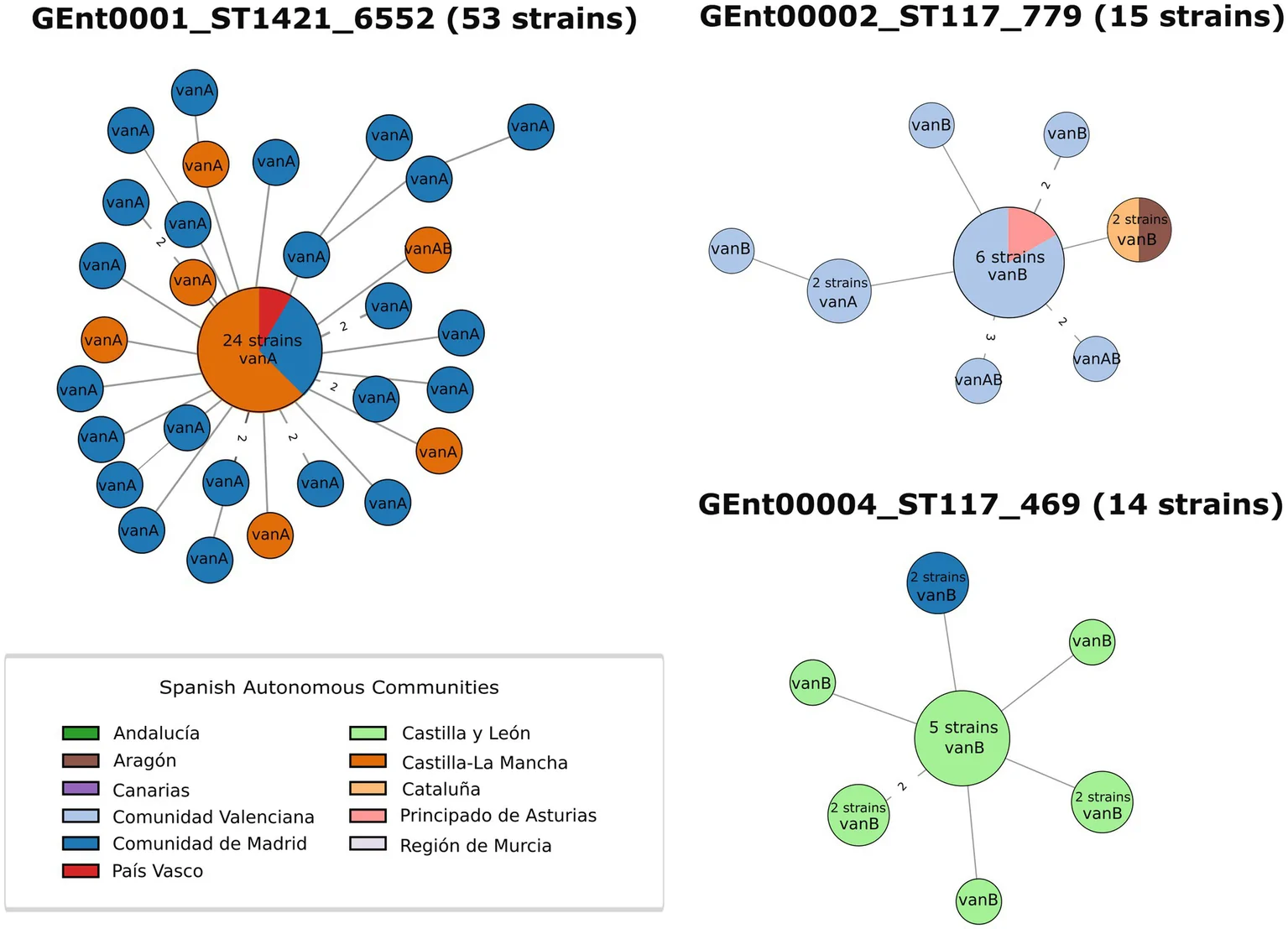
Main clusters of vancomycin-resistant *Enterococcus faecium* affecting three or more hospitals (cutoff point ≤5 alleles, cgMLST of 1,423 genes). A minimum spanning tree is shown with numerical indication only at distances greater than 1, and the circles are weighted according to the number of isolates up to a maximum of 6. The colour of the circle shading indicates the autonomous community in which the sample was taken. The number inside the circle indicates the number of isolates. In the cluster names, the number following the ST indicates the corresponding CT (cgMLST complex type).

Four clusters with *van*A*+van*B genotype isolates were identified; in two of them, most isolates showed this profile (GEnt0007 and GEnt0006, Figure 4), whereas in the other two it was minor (GEnt0001 and GEnt0002, Figure 3). The vanA*+van*B genotype isolates in clusters GEnt0007 and GEnt0006 affected more than one hospital within a single autonomous community.

**Figure 4:**
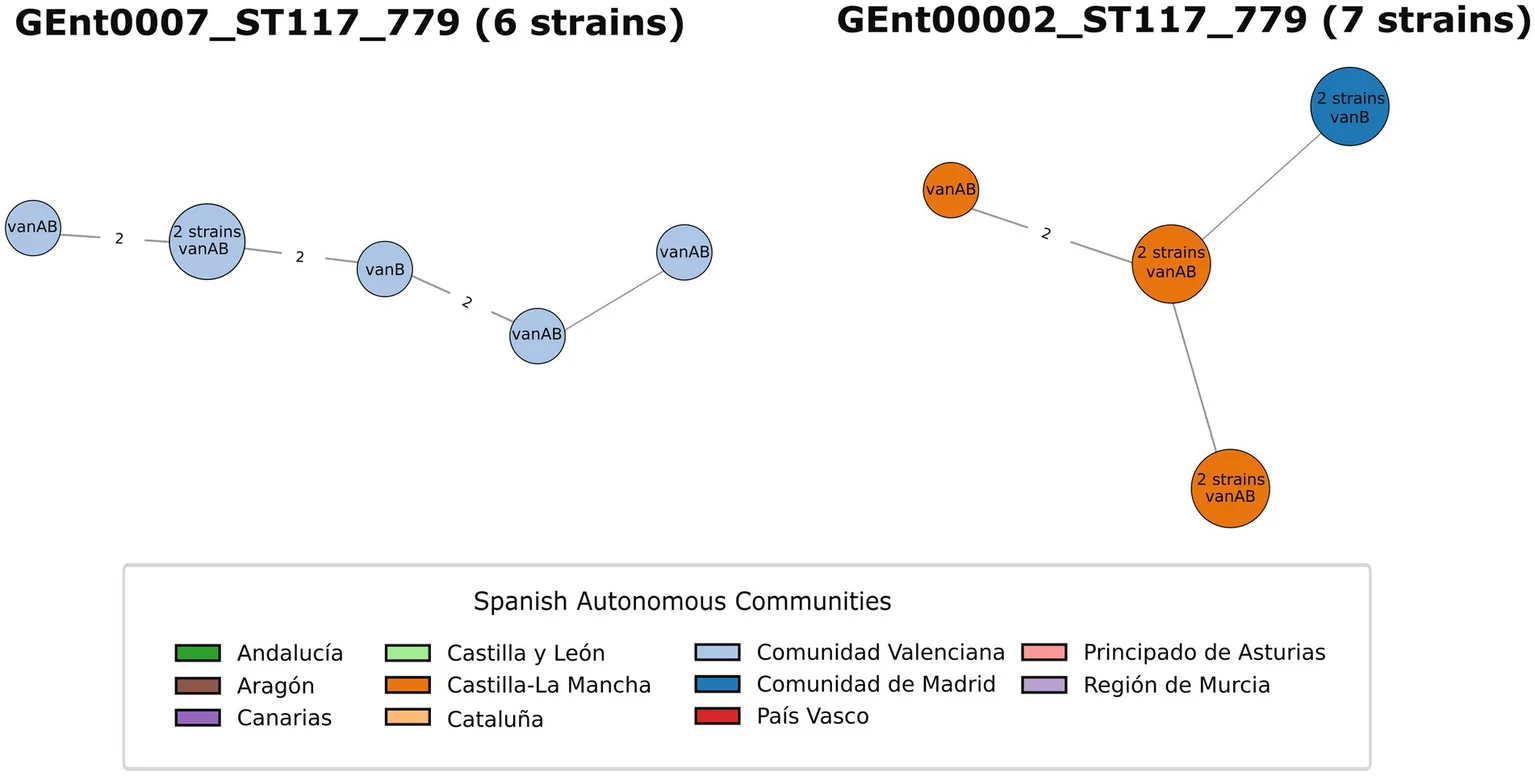
Clusters of vancomycin-resistant *Enterococcus faecium* exhibiting *van*A+*van*B (cutoff point ≤5 alleles, cgMLST of 1,423 genes). A minimum spanning tree is shown with numerical indication only at distances greater than 1, and the circles are weighted according to the number of isolates up to a maximum of 6. The colour of the circle shading indicates the Autonomous Community in which the sample was taken. The number inside the circle indicates the number of isolates. In the cluster names, the number following the ST indicates the corresponding CT (cgMLST complex type).

### Mechanisms of linezolid resistance in VREfm isolates

Among the 317 VREfm isolates selected for WGS, 51 (16.1%) also carried linezolid resistance mechanisms (Table 3). Of them, 25 (49%) were colonizations and 26 (51%) were infections, half of which were bloodstream infections. The cases were predominantly male (56.9%), over 65 years of age (60.8%), and were admitted to 15 hospitals across 8 Spanish provinces. The highest number of cases was detected in the provinces of Valencia (*n* = 22, from four hospitals) and Madrid (*n* = 16, from three hospitals).

**Table 3:** Characteristics of the 51 *Enterococcus faecium* isolates carrying vancomycin and linezolid resistance genes.

Linezolid resistance mechanism	*van*A	*van*B	*van*A *+van*B	Sequence types	Autonomous communities	Total
*optr*A+*cfr*D	21	1	9	ST117 (20), ST80 (10), ST17 (1)	Comunidad Valenciana (22), Comunidad de Madrid (9)	31
23S G2576T mutation	4	7	0	ST117 (6), ST80 (3), ST17 (1), ST761 (1)	Castilla León (4), Comunidad de Madrid (4), Comunidad Valenciana (1), Cataluña (1), Aragón (1)	11
*optr*A	3	1	0	ST117 (3), ST80 (1)	Comunidad Valenciana (3), Comunidad de Madrid (1)	4
*poxt*A	3	0	0	ST80 (1), ST1421 (1), ST1424 (1)	Comunidad de Madrid (2), Cataluña (1)	3
*cfr*D	0	0	1	ST117	Comunidad Valenciana	1
*optr*A+23S G2576T mutation	1	0	0	ST1380	Cataluña	1

Thirty-two (62.8%) isolates carried only *van*A gene, nine (17.6%) only *van*B, and 10 (19.6%) carried both the *van*A and *van*B genes. Regarding linezolid resistance mechanisms, *optr*A was the most frequently detected gene (36, 70.6%), followed by *cfr*D (32, 62.7%) and *poxt*A (3, 5.9%). A strong association between *optr*A and *cfr*D was observed (*n* = 31, 60.8%) (OR = 1,736.0, IC (95%) = 196.5–15,340.2, *p* < 0.0001). The G2576T mutation in the 23S rRNA subunit was detected in 12 isolates (23.5%) applying a 10% threshold (26).

Isolates carrying the *optr*A*/cfr*D genes predominantly harbored the *van*A genotype, either with (9/31, 29%) or without (21/31, 67.7%) the additional *van*B gene, whereas isolates with the G2576T mutation in the 23S rRNA subunit mainly belonged to the *van*B genotype (7/12, 58.3%).

MLST analysis revealed seven STs among the 51 isolates with vancomycin and linezolid resistance mechanisms, all belonging to clonal complex 17 (CC17). However, very low ST diversity was observed, with two STs accounting for 88.2% of all isolates: ST117 (30, 58.8%) and ST80 (15, 29.4%).

The 30 isolates carrying the *van*A, *optr*A, and *cfr*D genes, with or without *van*B, belonged to ST117 (63.3%), ST80 (33.3%), or ST17 (3.3%), and were recovered from seven hospitals across three provinces (Valencia, Madrid, and Castellón). The 33.3% (10/30) of these isolates was involved in invasive infections, although no statistical differences were observed compared with the invasive infections produced by the rest of the sequenced isolates (62/287, 21.6%) (OR = 1.9, IC (95%) = 0.8–4.2, *p* = 0.1358).

## Discussion

The implementation of well-coordinated control measures at different levels is key to controlling multidrug resistance to antibiotics. However, for these measures to be effective and cost-efficient, they must be guided by prior knowledge of the problem; knowledge that is as up-to-date, accurate, and comprehensive as possible. To this end, it is necessary to develop holistic, integrated, and high-quality AMR surveillance systems that enable the early analysis of combined data from different sources, thereby guiding decision-making for the control of AMR. In particular, the European Centre for Disease Prevention and Control (ECDC) promotes the integration of genomic sequencing into the surveillance of multidrug-resistant pathogens through the Strategic Framework developed on this subject (1).

RedLabRA was officially established in Spain with this objective, within the framework of the National Plan against Antibiotic Resistance (PRAN), and with the approval of the Interterritorial Council and the Public Health Commission of the Spanish National Health System (27). Although the network prioritizes the continuous surveillance of carbapenemase-producing *Klebsiella pneumoniae*, *Enterobacter cloacae* complex, and *Escherichia coli*, it is designed and equipped to respond to changing epidemiological trends, as demonstrated by the emergence of vancomycin-resistant enterococci (VRE), which are the focus of the present study.

RedLabRA is structured to function as a national network comprised of regional sub-networks, ensuring that all microbiology laboratories in the country are included. However, the heterogeneity of the different autonomous communities means that the network’s implementation in some of them has not yet reached full capacity, and therefore some hospitals may remain outside of RedLabRA’s current coverage.

This study highlighted several noteworthy findings, including: (i) the ability of RedLabRA to investigate emerging threats in antimicrobial resistance through genomic analysis, regardless of whether they are included among routinely monitored surveillance targets, (ii) the inter-hospital and inter-regional presence of VREfm isolates with high genetic similarity belonging to the STs ST117, ST80, and ST1421, and (iii) the emergence and spread of VREfm isolates harboring resistance mechanisms to both vancomycin and linezolid, most notably the *vanA* + *optrA* + *cfrD* gene combination.

It is worth noting that the inclusion criteria for sequencing were designed to achieve the greatest possible representation of geographic areas, clones, and resistance mechanisms, as well as to avoid overrepresentation due to outbreaks and/or endemics; prioritizing the ability to detect possible spreads between regions and hospitals. By that, they sequenced isolates do not necessarily reflect the real prevalence of the clones and resistance mechanisms.

The low number of vancomycin-resistant *E. faecalis* cases detected led to the genomic study focusing solely on *Efm*. Recent data from EARS-Net, although only collecting data from blood isolates, show vancomycin resistance rates of 0.1 and 2.6% in 2024 for *E. faecalis* and *Efm*, respectively (10, 28).

Historical VRE data collected by EARS-Net have remained below 5% in Spain for Efm. However, higher rates have been reported in neighbouring countries such as Portugal, Greece, and Italy (28). The high prevalence in these countries emphasizes the considerable risk of escalation of this antimicrobial resistance public health threat. This marked geographical variability observed among European countries has also been identified in this study across different Spanish regions. Up to 8 autonomous communities, as well as Ceuta and Melilla, were identified as having no cases, or only sporadic cases, of VRE; however, five reported a high number of cases and had between 7 and 14 affected hospitals. In recent years, several VRE outbreaks have been reported in Spain, predominantly involving carriers rather than infected patients (29–32).

ST1421, identified as an emerging lineage in Spain in this study, has previously been associated with shifts in VRE populations in other countries such as Denmark (33) and Australia (34). The other two predominant STs in this study, ST117 and ST80, have also recently been linked to marked increases in VRE prevalence in Germany (35) and Ireland (36), respectively.

The accumulation of antibiotic resistance mechanisms in a single bacterial strain is a major concern, as it drives the emergence of extensively drug-resistant or pandrug-resistant bacteria. The emergence of *Efm* isolates harboring both vancomycin resistance genes (*van*A and *van*B) as well as acquired resistance genes to linezolid, one of the main alternative therapeutic options to glycopeptides, detected in this study is particularly noteworthy. The co-location of the *van*A, *optr*A, and *cfr*D genes on a single linear plasmid has recently been reported in various isolates of both *Efm* and *E. faecalis* (14, 37). Regarding this issue, it is worth considering that the presence of genes or mutations encoding resistance to linezolid does not always reflect, although it does in a majority way, phenotypic resistance to this antibiotic.

It is noteworthy the high frequency of invasive infections caused by *Efm* isolates carrying *van*A*+ optr*A*+cfr*D, in contrast to what is observed for the overall population of VREfm sequenced isolates, a finding that requires close monitoring.

In addition to some already mentioned previously, this work has the following limitations: (i) the lack of availability of population data for calculating incidences and (ii) the genomic sequencing of only a sample of the total cases detected in the study period, which does not allow drawing general conclusions about the total national prevalence of clones and mechanisms.

In conclusion, this study promoted by RedLabRA in response to the increase in VRE cases in certain Spanish autonomous communities has revealed a complex and evolving scenario that requires close monitoring to minimize the risk of VRE reaching the prevalence levels observed in neighbouring European countries.

## Data Availability

The datasets presented in this study can be found in online repositories. The names of the repository/repositories and accession number(s) can be found at: https://www.ebi.ac.uk/ena, PRJEB123727.

## Footnotes

1

2
https://www.cgmlst.org/ncs

3
https://docs.scipy.org/doc/scipy/reference/cluster.hierarchy.html
