# Supplementary material for: Vancomycin-resistant enterococci in Spanish hospitals: the experience of official network for the surveillance of resistant microorganisms RedLabRA, 2023–2024
Source: Front Public Health. 2026 Oct 29;14:1966902. doi: 10.3389/fpubh.2026.1966902 (PMC13755271; doi:10.3389/fpubh.2026.1966902)
Supplement: Supplementary file 1 [file Supplementary_file_1.pdf]

# Appendix 1 – Members of the Spanish RedLabRA Network Study Group per Autonomous Community:

**Andalucía**

**Hospital Universitario Virgen Macarena**: Lorena López Cerero, Álvaro Pascual Hernández.

**Aragón**

**Hospital Clínico Universitario Lozano Blesa**: Jessica Bueno, Cristina Seral.

**Hospital Universitario Miguel Servet**: Ana Isabel López Calleja, Ana Milagro, Blanca Fortuño, Alexander Tristancho, Marta Gómara, Concepción López.

**Asturias**

**Hospital Universitario Central de Asturias**: Javier Fernandez Domínguez, Carlos Rodríguez Lucas.

**Cantabria**

**Hospital Universitario Marqués de Valdecilla**: Jorge Calvo Montes, Domingo Fernández Vecilla, Sergio García Fernández.

**Castilla y León**

**Complejo Asistencial Universitario de Burgos**: María Pilar Ortega Lafont, María Isabel Andrés Franch.

**Hospital Universitario Río Hortega**: Luís López-Urrutia Lorente, Irene Merino Velasco.

**Castilla-La Mancha**

**Hospital Universitario de Guadalajara**: Alejandro González Praetorius, Sara Pérez de Madrid.

**Cataluña**

**Hospital Clínic de Barcelona**: Cristina Pitart, Ignasi Roca, Jordi Vila.

**Hospital Santa Creu i Sant Pau**: Elisenda Miró, Ferran Navarro Risueño, Alba Rivera.

**Hospital Universitario de Bellvitge**: Jordi Cámara, Aida González-Díaz, Manuel González de Aledo, María Ángeles Domínguez Luzón.

**Hospital Universitario Vall d'Hebron**: Juan José González López, Nieves Larrosa Escartín, Alba Mir, Albert Moreno Mingorance, Guillem Puigsech Boixeda.

__Ceuta__

**Hospital Universitario de Ceuta**: Patricia González Donapetry.

__Comunidad de Madrid__

**Hospital Clínico San Carlos**: Alberto Delgado-Iribarren, Fernando González Romo, Paloma Merino Amador.

**Hospital General Universitario Gregorio Marañón:** Mercedes Marín Arriaza, Patricia Muñoz García, Julia Serrano Lobo.

**Hospital Universitario 12 de octubre**: Beatriz González, Esther Viedma Moreno, Jennifer Villa García.

**Hospital Universitario la Paz**: Julio García Rodríguez, Fernando Lázaro Perona.

**Hospital Universitario La Princesa**: Teresa Alarcón Cavero, Laura María Cardeñoso, María Díez Aguilar.

**Hospital Universitario Puerta de Hierro**: Alberto Nieto Fernández, Maria Isabel Sánchez Romero.

**Hospital Universitario Ramón y Cajal**: Rafael Cantón, Marta Hernández García, Pedro Manuel Ponce Alonso, Patricia Ruiz Garbajosa**.**

__Comunidad Foral de Navarra__

**Hospital Universitario de Navarra**: Maddi Olazábal, Eugenia Portillo, Iosu Razquin.

__Comunidad Valenciana__

**Consorcio Hospital General Universitario de Valencia**: Concepción Gimeno Cardona, Rafael Medina, Nuria Tormo.

**Hospital Clínico Universitario de Valencia**: David Navarro Ortega, Javier Colomina, Estela Giménez, Ignacio Torres Fink.

**Hospital General Universitario de Alicante – Balmis**: Juan Carlos Rodríguez Díaz, María Paz Ventero.

**Hospital General Universitario de Castellón**: Mª Dolores Tirado Balaguer, Susana Sabater Vidal, Bárbara Gomila Sard.

**Hospital General Universitario de Elche:** Nieves Gonzalo Jiménez, Antonio Galiana Cabrera

**Hospital Universitario Doctor Peset**: Juan J. Camarena Miñana, Macarena Pariente Martín.

**Hospital Universitario y Politécnico La Fe**: Mª Dolores Gómez, Salvador Giner Alcaraz, Eva María González Barberá, Noelia Lozano.

**FISABIO:** Fernando González Candelas, Carlos Valiente Mullor, Lidia Ruiz Roldán, Alejandro Sanz, Regina Antoni.

**Extremadura**

**Complejo Hospitalario Universitario de Cáceres**: Cristina García Pérez, Helena Gil Campesino, María Izquierdo de Miguel.

**Hospital Universitario de Badajoz**: Miguel Fajardo Olivares, Cristina Gaona.

**Galicia**

**Complejo Hospitalario Universitario de A Coruña**: Jorge Arca, Germán Bou Arévalo, Ana Fernández González.

**Complejo Hospitalario Universitario de Santiago**: Antonio Aguilera, José Javier Costa Alcalde, Adrián Domínguez Lago, Alberto Morales Vila, Daniel Navarro de la Cruz, Noelia Parajó Pazos.

**Complejo Hospitalario Universitario de Vigo**: Sonia Pérez Castro, Sonia María Rey Cao, Francisco José Vasallo Vidal.

**Islas Baleares**

**Hospital Universitario Son Espases**: María Fernández-Billón Castrillo, Carla López, Antonio Oliver Palomo.

**Islas Canarias**

**Hospital Universitario Nuestra Señora de Candelaria**: Julia Alcoba Florez, Diego García Martínez de Artola.

**La Rioja**

**Hospital San Pedro**: Carla Andrea (Andrea) Alonso Arribas, José Manuel Azcona Gutiérrez, Mirian Blasco Alberdi, Estíbaliz Erviti Machain.

**Melilla**

**Hospital Comarcal de Melilla**: Sergio Román.

**País Vasco**

**Hospital Universitario Basurto**: José Luis Díaz Tuesta del Arco, Estíbaliz Ugalde, Matxalen Vidal García.

**Hospital Universitario de Araba, Txagorritxu**: Jorge Arribas García, Andrés Canut.

**Hospital Universitario de Cruces**: José Luis Barrios, Clara Lejarraga Cañas, Leyre Mónica López Soria.

**Hospital Universitario Donostia**: José Mª Marimón, Diego Vicente Anza.

**Región de Murcia**

**Hospital Clínico Universitario Virgen de la Arrixaca**: Teresa García, Laura Moreno Parrado, Marina Simón, Genoveva Yagüe Guirao.
