# Supplementary material for: Vancomycin-resistant enterococci in Spanish hospitals: the experience of official network for the surveillance of resistant microorganisms RedLabRA, 2023–2024
Source: Front Public Health. 2026 Oct 29;14:1966902. doi: 10.3389/fpubh.2026.1966902 (PMC13755271; doi:10.3389/fpubh.2026.1966902)
Supplement: Supplementary file 2 [file Supplementary_file_2.pdf]

Supplementary Material – Appendix 2.

Questionnaire sent to RedLabRA Level 2 Laboratories on cases of vancomycin-resistant enterococci

---

1. Name of the Level 2 Laboratory (LN2) to which you belong: *

Write your answer.

2. Contact email address: *

Write your answer.

---

3. Number of cases detected in 2023 within your LN2 and the LN1 laboratories you coordinate: *

(Total number of cases from all coordinated LN1 laboratories)

The value must be a number.

---

4. 2023 Cases – Free text:

(Only from your hospital? From how many hospitals? etc.)

Write your answer.

---

5. Number of cases detected in 2024 within your LN2 and the LN1 laboratories you coordinate: *

(Total number of cases from all coordinated LN1 laboratories)

The value must be a number.

---

6. 2024 Cases – Free text:

(Only from your hospital? From how many hospitals? etc.)

Write your answer.

---

7. General impression of the issue:

(Free text: identified trends, response to the problem, etc.)

Write your answer.

---

8. Personal assessment of the current priority of this resistance problem in your area, based on its clinical and epidemiological impact:

**Priority Level**	**Selection**
Critical	☐
High	☐
Medium	☐
Low	☐
Not applicable	☐
